# Associations of gestational age with gyrification and neurocognition in healthy adults

**DOI:** 10.1007/s00406-022-01454-0

**Published:** 2022-07-29

**Authors:** Simon Schmitt, Kai G. Ringwald, Tina Meller, Frederike Stein, Katharina Brosch, Julia-Katharina Pfarr, Tim Hahn, Hannah Lemke, Susanne Meinert, Jonathan Repple, Katharina Thiel, Lena Waltemate, Alexandra Winter, Dominik Grotegerd, Astrid Dempfle, Andreas Jansen, Axel Krug, Udo Dannlowski, Igor Nenadić, Tilo Kircher

**Affiliations:** 1 Department of Psychiatry and Psychotherapy, Philipps University of Marburg, Rudolf-Bultmann-Str. 8, 35039 Marburg, Germany; 2 Center for Mind, Brain and Behavior (CMBB), University of Marburg and Justus Liebig University Giessen, Marburg, Germany; 3 Marburg University Hospital – UKGM, Marburg, Germany; 4 Institute for Translational Psychiatry, University of Münster, Münster, Germany; 5 Core-Facility BrainImaging, Faculty of Medicine, Marburg, Germany; 6 Institute of Medical Informatics and Statistics, University Hospital Schleswig-Holstein, Kiel, Germany; 7 Department of Psychiatry and Psychotherapy, University of Bonn, Bonn, Germany; 8 Department of Psychiatry, Social Psychiatry and Psychotherapy, Hannover Medical School, Hannover, Germany

**Keywords:** Gyrification, Gestational age, Prenatal brain development, Cognition, Language, Working memory

## Abstract

Epidemiological studies have shown that gestational age and birth weight are linked to cognitive performance in adults. On a neurobiological level, this effect is hypothesized to be related to cortical gyrification, which is determined primarily during fetal development. The relationships between gestational age, gyrification and specific cognitive abilities in adults are still poorly understood. In 542 healthy participants, gyrification indices were calculated from structural magnetic resonance imaging T1 data at 3 T using CAT12. After applying a battery of neuropsychological tests, neuropsychological factors were extracted with a factor analysis. We conducted regressions to test associations between gyrification and gestational age as well as birth weight. Moderation analyses explored the relationships between gestational age, gyrification and neuropsychological factors. Gestational age is significantly positively associated with cortical folding in the left supramarginal, bilaterally in the superior frontal and the lingual cortex. We extracted two neuropsychological factors that describe language abilities and working memory/attention. The association between gyrification in the left superior frontal gyrus and working memory/attention was moderated by gestational age. Further, the association between gyrification in the left supramarginal cortex and both, working memory/attention as well as language, were moderated by gestational age. Gyrification is associated with gestational age and related to specific neuropsychological outcomes in healthy adulthood. Implications from these findings for the cortical neurodevelopment of cognitive domains and mental health are discussed.

**Supplementary Information:**

The online version contains supplementary material available at 10.1007/s00406-022-01454-0.

## Introduction

Epidemiological studies have shown that aspects of fetal growth such as birth weight and gestational age are positively correlated with cognitive abilities in adults [1–6]. The brain morphological mechanisms underlying these effects are not yet fully understood.

The fetus is constantly exposed to environmental effects [7]. For example, maternal stress [8, 9], anxiety [10, 11], smoking [12], malnutrition [13] and social status [14] are associated with lower birth weight and gestational age. Therefore, birth weight and gestational age served as surrogate markers for a large variety of environmental influences on prenatal development in many studies [1].

Gyrification is an index measuring cortical folding which can be extracted from MRI images. This measure has been utilized in imaging studies investigating the course of cortical development in utero, in new-borns, and throughout life [15, 16]. Reduced birth weight or shortened gestational age leads to increases and decreases in gyrification distributed over large parts of the cortex, but especially in fronto-temporo-parietal regions [17, 18]. Consistent with the current conceptualization of gyrification as an index largely determined during intrauterine development [19–21], these structural alterations persist over the whole life span and can still be found in adults. Further, some of these gyrification alterations associated with prenatal growth have been linked to neurocognitive performance in infants and adults [17, 18, 22]: Full-scale IQ has been associated with the left fusiform gyrus and lateral orbitofrontal cortex, the right superior parietal gyrus [18], the bihemispheric lateral and anterior temporal cortices and the occipitotemporal junction [17]. Regional variations in gyrification have repeatedly been associated with general neurocognitive performance in humans [23, 24] and are considered as a potential neurocellular correlate of cognitive abilities.

While these studies have advanced our knowledge considerably, they leave several questions unanswered: 1. Many of the previous studies used only group comparisons (with arbitrary criteria for the definition of cut-offs for group divisions that were not based on neurobiological measures), rather than dimensional approaches, resulting in a loss of variance. 2. Most studies used only moderate sample sizes leading to little statistical power and limited generalizability. 3. Cortical folding takes place in a non-linear course primarily during the second half of intrauterine development but also non-linearly ex utero in infants [1, 25–27]. However, previous studies did not test for non-linear associations between variables of prenatal growth and brain morphology. 4. Some studies included participants with adverse events during their gestation such as birth complications, maternal infection, alcohol or drug abuse, medication, or maternal malnutrition [15, 17, 18, 28, 29], which all confound birth weight/gestational age as well as brain structural alterations. 5. Lastly, most studies investigated only intelligence by using abbreviated tests and refrained from measuring a broad range of cognitive domains. Some also used merely subjective parental reports as a proxy of child cognitive abilities. As a result, effects on specific cognitive domains, e.g., working, short- and long-term memory, attention or language have yet to be investigated.

In our study, we aimed to address these points. We recruited a large sample of healthy participants but excluded all subjects born after a high-risk pregnancy who had been exposed to gross harmful environmental influences, e.g., maternal infections, drug use, malnutrition or birth complications. Further, we used a comprehensive neuropsychological test battery to investigate associations between gyrification, fetal growth and specific neuropsychological domains. We hypothesized: 1. Gestational age as well as 2. birth weight are associated with reduced gyrification in healthy adults. 3. These relationships are non-linear. 4. Finally, we test whether gyrification is related to neuropsychological performance in adults, thus bridging the explanatory gap between prenatal development and adult neuropsychological outcomes.

## Materials and methods

### Sample

Healthy participants were recruited from the ongoing bicentric FOR2107 study (http://for2107.de/; [30]). All subjects underwent the Structured Clinical Interview (SCID-I; [31]) based on the DSM-IV-TR which was administered by trained researchers to ensure absence of current or history of any psychiatric disorders over life time. Additional exclusion criteria were an IQ < 80 (estimated with the MWT-B [32], a German equivalent of the National Adult Reading Test (NART; [33])) or age > 65 years or < 18 years. Further, participants with current or previous substance dependence, major medical conditions (e.g., cancer, chronic autoimmune diseases, infections) and any history of neurological diagnoses (stroke, tumor, neuro-inflammatory diseases, epilepsy, head-trauma) were excluded. All participants with contraindications to MRI (e.g., pregnancy, ferromagnetic implants, claustrophobia), were also excluded from this study.

Based on the literature, we identified different types of high-risk pregnancies that could confound our results and excluded all subjects born after such high-risk pregnancies. These risk factors were: maternal infections which are associated with increased chance for long-term cognitive deficits [34] and mental retardation [35] and alcohol consumption by the mother during pregnancy, which is associated with behavioral problems, cognitive deficits and increased stress reactivity [36–38]. Additionally, we excluded participants whose mothers used drugs during pregnancy which is also associated with higher risk for behavioral problems and cognitive deficits [39–43]. Another exclusion criterion was maternal malnourishment which substantially affects fetal brain development [15, 44] that may have long-term consequences for behavioral problems and cognitive function later in life [45–49]. Multiple births were also excluded because fetal growth restrictions are associated with a decrease in cortical folding [50, 51], which is more common in multiple pregnancies [52]. Last, subjects who were born with severe obstetric complications were also excluded, e.g., ischemia, which is associated with an increase for both mental disorders [53, 54] as well as brain structural alterations in cortical volumes and total surface areas [55] and a decrease in intelligence [56]. Detailed information on the course of pregnancy and the presence of a high-risk pregnancy, including gestational age and birthweight, were obtained via questionnaires. These were sent to the participants via mail two weeks before the appointment for MRI examination, so that they had enough time to obtain this information, e.g., from their birth certificate.

The final sample consisted of 542 nonclinical participants. Their birth weight ranged from 1000 to 5300 g, their gestational age from 28 to 42 weeks. Birth weight and gestational age were correlated (*r* = 0.378; *p* < 0.001). Based on the WHO definition of preterm birth (being born before the 38th week of gestation [57]), 51 subjects from the 542 participants were born premature. 367 participants (67.7%) were female, the mean age of the sample was 31.6 years (*SD* = 11.5 years) and, on average, participants had 13.93 years of education (*SD* = 2.4). Characteristics of participants are listed in Table 1.

**Table 1:** Sociodemographic characteristics and neuropsychological test scores

	Age	Sex	Gestational age	Birth Weight (g)	Education (years)	Handedness	Neuropsychological test scores										
							VLMT-A	VLMT-B	TMT-B	Corsi	LNST	DSST	d2-KL	RWT-A	RWT-P	RWT-Alt	MWTB
Mean	31.6	*f* = 367	39.57	3416	13.93	77.27	60.05	0.81	47.12	18.31	17.18	65.52	193.3	25.1	12.32	16.71	30.85
SD	11.5	–	1.63	528.2	2.447	41.85	8.02	1.52	17.04	3.07	3.05	11.45	41.07	5.54	4.13	2.96	2.933
Minimum	18	–	28	1000	9	− 100	28	− 3	14	9	7	7	79	3	2	7	17
Maximum	64	–	42	5300	18	100	75	8	173	26	24	93	298	44	26	30	37

Handedness was assessed using the EHI [110]. *f* female, *VLMT* verbal learning and memory test (VLMT A short-term memory, VLMT B long-term memory), *TMT-B* trailmaking test, *Corsi* Corsi block tapping task, *LNST* letter–number sequencing subtest, *DSST* digit symbol substitution test, *d2-KL* sustained-attention test d2, *RWT* Regensburger Wortflüssigkeits-Test (versions: *A*: category animals; *P*: words that start with the letter 'p'; alt: alternating between fruits and sports), *MWTB* Multiple choice vocabulary test

### MRI data acquisition

We acquired MRI data at two sites and in accordance with our quality assurance protocol [58]. In Marburg, the MRI data were acquired with a 3 T MRI scanner (Tim Trio, Siemens, Erlangen, Germany) using a 12-channel head matrix Rx-coil. In Münster, a 3 T MRI scanner (Prisma, Siemens, Erlangen, Germany) and a 20-channel head matrix Rx-coil were used. Parameters of the MP-RAGE sequences differed slightly across sites (Münster: TR = 1900 ms, TE = 2.28 ms, Flip angle = 8°, 192 sagittal slices, voxel size 1 × 1x1 mm, acquisition duration = 4:58 min; Marburg: TR = 1900 ms, TE = 2.26 ms, Flip angle = 9°, 176 sagittal slices, voxel size 1 × 1 × 1 mm, acquisition duration = 4:26 min). Precluding their further preprocessing, scans were manually checked for the absence of artifacts and inspected for anatomical abnormalities by a senior clinician (UD). In doing so, we excluded scans from six participants.

### MRI data preprocessing and statistical analysis of gyrification data

For surface based morphometry analyses, we used the CAT12 toolbox (version r1450) that builds on SPM [59]. Applying default settings, we extracted cortical surfaces using a spherical harmonics approach [60], applied topological correction [61] and mapped surfaces spherically with an adopted volume-based diffeomorphic DARTEL algorithm [62] to reparametrize them into a common coordinate system to allow inter-subject comparisons [63]. We then estimated cortical gyrification utilizing an approach based on local cortical absolute mean curvature (AMC; [64]). Increases in the amplitude and frequency of the folding of the cortex are reflected in increased values of smoothed absolute mean curvature. All modulated gyrification data sets were smoothed with a 25 mm full width at half maximum Gaussian kernel. For cluster labeling, we chose the Desikan-Killiany-40 Atlas [65].

Separate polynomial general linear models were calculated with gestational age (in weeks) and birth weight, respectively, as regressors and gyrification as regressand. To investigate potential quadratic functions, we used the CAT12 function *cat_stat_polynomial* to estimate quadratic functions of the parameters birth weight and gestational age. Additionally, we used age, gender, site and scanner as covariates. Further, we conducted two-sample t-tests comparing participants born after full-term pregnancy *versus* participants born preterm regarding differences in gyrification with identical covariates.

Contrasts were processed using the TFCE Toolbox (threshold-free cluster enhancement; version r211) according to the Smith Method and with 8000 permutations [66–68]. We set significance levels at α < 0.05, family wise error corrected (FWE; [69, 70]). FWE-significant mean cluster raw values were extracted for each participant for further analyses (see below).

### Neuropsychological assessment

The neuropsychological assessment took about 50 min and contained eight different tests that measure verbal fluency (Regensburger Wortflüssigkeits-Test (RWT); [71]), verbal IQ (Mehrfachwahl-Wortschatztest (MWT-B); [32]), processing speed (Trail Making Test (TMT-B); 72, digit symbol substitution test (DSST); [73]), sustained-attention (sustained-attention test d2; [74]), declarative short- and declarative long-term memory (Verbaler Lern- und Merkfähigkeitstest (VLMT-A and VLMT-B; [75])) and working memory (Letter–number sequencing subtest (LNST; 73, Corsi; [76])). Complete neuropsychological data were available for 530 participants. For further information on the execution of the neuropsychological assessment and an overview of used test scores, see supplement; for a descriptive statistics of neuropsychological test scores, see Table 1.

We conducted an exploratory factor analysis with SPSS 24 [77] using the aforementioned neuropsychological test scores to describe variability among inter-correlated variables and generate a set of factors with reduced dimensionality. Factors were extracted by principal axis factoring and number of factors was determined using a scree test [78]. Maximizing loadings of variables on particular factors was ensured by conducting a varimax rotation. The Kaiser–Meyer–Olkin measure of sampling was 0.846, indicating the test measures sampling adequacy is given. Barlett’s test of Sphericity yielded a significant result (*p* < 0.001) suggesting that correlations between items were sufficiently large for performing a factor analysis. An anti-image correlation matrix revealed that all variables showed MSA-values (measure of sampling adequacy) > 0.7, implicating that they are all suitable for being used in a factor analysis (Table S1). Neuropsychological test scores intercorrelations revealed that none of them showed intercorrelations with the other variables always below 0.3, meaning that we did not include variables merely contributing to the understanding of the factor’s underlying data structure (Table S2). Additionally, no intercorrelation was above 0.9 suggesting no existence of multicollinearity (Table S2). For each subject, factor scores were computed utilizing a regression approach.

### Partial correlations between neuropsychological factors and gyrification

To investigate potential relationships between extracted mean gyrification from significant clusters and neuropsychological test outcome, we performed partial correlations while controlling for years of education. Brain structural alterations are much more pronounced in humans born premature [26, 79–82]. Therefore, we performed partial correlations separating participants into two groups: born preterm (as defined as gestational age shorter than 38 weeks by the WHO [57]) and born from the 38th week of gestation or later.

### Moderation analyses

We conducted moderation analyses using the PROCESS macro 3.5 [83] available for SPSS to investigate contributions of gestational age on the gyrification–gestational age associations and neuropsychological performances. Raw mean gyrification values were extracted from significant clusters of vertices and subsequently used as our predictors, the two extracted neuropsychological factors as outcome variables, gestational age in weeks as moderator and, additionally, years of education [84] as a covariate. Heteroscedasticity consistent standard error and covariance matrix estimator was used (HC3) [85, 86] and continuous variables that define products were mean-centered prior to analysis. Due to the negative skewness of the variable gestational age (skewness = − 2) moderations were probed at the 16th, 50th and 84th percentiles.

### Ethics

The FOR2107 cohort project was approved by the Ethics Committees of the Medical Faculties, University of Marburg (AZ: 07/14) and University of Münster (AZ: 2014-422-b-S). The authors assert that all procedures contributing to this work comply with the ethical standards of the relevant national and institutional committees on human experimentation and with the Helsinki Declaration of 1975, as revised in 2008. All subjects gave written informed consent to our study protocol and received financial compensation for their expense.

## Results

### Gyrification

**Fig. 1:**
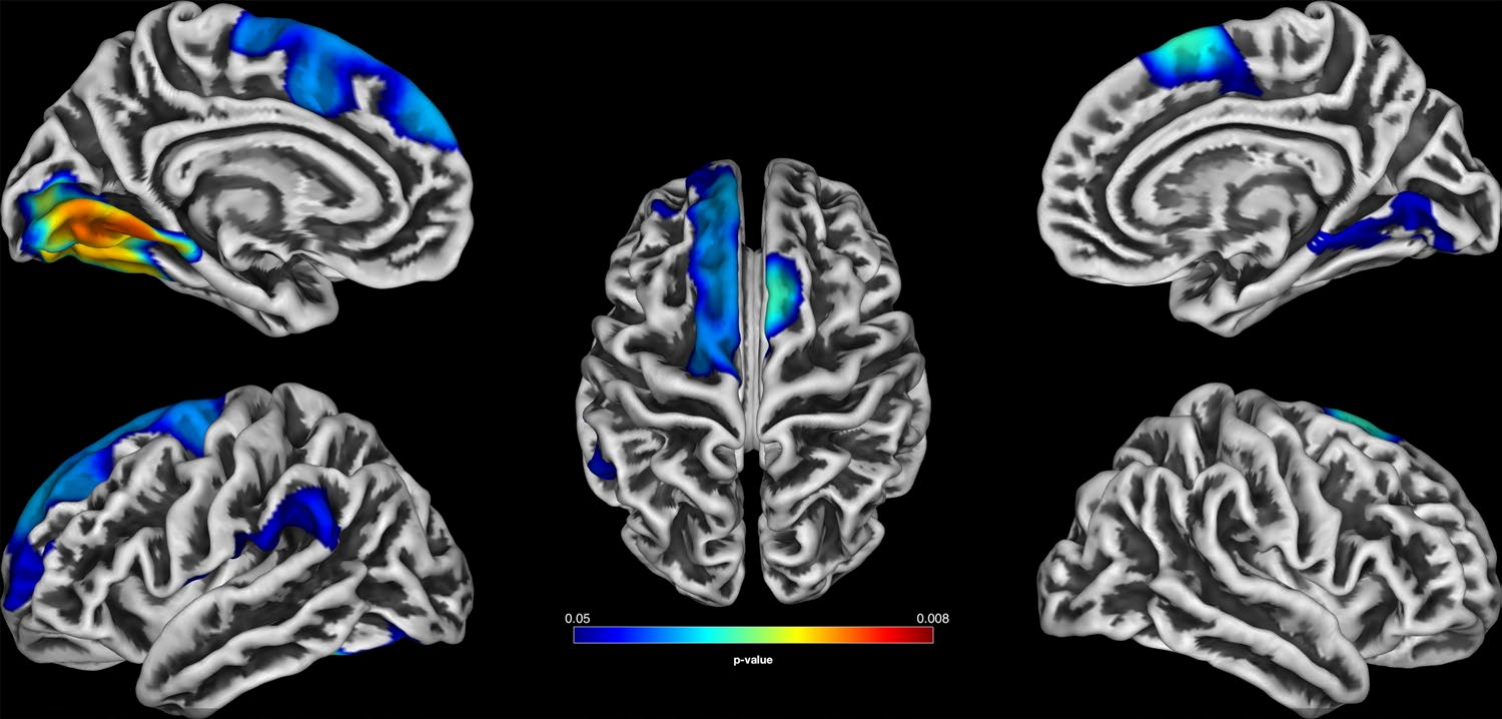
Associations between gyrification and gestational age. Statistical parametric map of positive associations between gestational age (in weeks) and gyrification in a multiple regression. Threshold-free cluster enhancement was used at a threshold of *p* < 0.05 (FWE-corrected). Warmer colors represent lower *p* values

We found significant positive linear associations between gestational age (*mean* = 39.57 weeks; *SD* = 1.63 weeks) and five gyrification clusters (s. Fig. 1, Table 2 and Fig. S1 for scatter plots) mainly involving the left supramarginal cortex (*k* = 881, TFCE = 12,398.62, *p*_FWE_ = 0.036), the left (*k* = 1978, TFCE = 13,555.62, *p*_FWE_ = 0.028) and right superior frontal cortex (*k* = 566, TFCE = 15,217.21, *p*_FWE_ = 0.019), and both the left (*k* = 1786, TFCE = 17,795.10, *p*_FWE_ = 0.01) and right lingual cortex (*k* = 671, TFCE = 12,027.61, *p*_FWE_ = 0.04). We found neither negative linear nor positive and negative quadratic associations between duration of pregnancy and gyrification. Conducted t-tests showed similar results. Participants born preterm showed relatively less gyrification compared to those born after full-term pregnancy in two significant clusters (*k* = 1057, TFCE = 15,713.68, *p*_FWE_ = 0.014; *k* = 7591, TFCE = 15,199.91, *p*_FWE_ = 0.016) that again included the bilateral superior frontal cortex, the left supramarginal cortex but not the lingual cortex (see Table S3 and Fig. S2). There were no brain areas in which participants born preterm showed significant more gyrification compared to participants born after full-term pregnancy. Birth weight (mean = 3416 g; *SD* = 528.2 g) was not significantly associated with gyrification in any of the regressions.

**Table 2:** Positive linear associations between duration of pregnancy in weeks and gyrification in a polynomial regression analysis

*k*	TFCE	*p*_FWE_	Coordinates	%	Anatomical region
1978	13555.62	0.028	-08/-11/64	80	l. superior frontal
	11909.78	0.041	-23/56/06	14	l. rostral middle frontal
	11883.15	0.041	-21/61/13	3	l. precentral
				2	l. paracentral
				2	l. caudal anterior cingulate
1786	17795.10	0.010	-29/-68/-14	44	l. lingual
	14328.93	0.023	-22/-35/-09	22	l. fusiform
				15	l. pericalcarine
				10	l. lateral occipital
				7	l. parahippocampal
				2	l. cuneus
881	12398.62	0.036	-56/-54/22	63	l. supramarginal
				21	l. postcentral
				14	l. precentral
671	12027.61	0.040	22/-73/-09	61	r. lingual
	11744.86	0.043	20/-82/-13	22	r. parahippocampal
				15	r. fusiform
				1	r. lateral occipital
566	15217.21	0.019	08/15/65	98	r. superior frontal
				1	r. posterior cingulate

Correction for multiple testing was carried out with the family wise error rate (FWE) at α = 0.05. Cluster labeling was conducted with the Desikan-Killiany atlas [65]

### Factor analysis of neuropsychological test scores

Based on the “elbow criterion” of a scree test (see Fig. S3) we retained two factors [78] that explained in total 36.2% of the variance in the neuropsychological data. The first one is mainly comprising neuropsychological tests measuring working memory and attention (eigenvalue = 3.46; with high rotated factor loadings on d2-KL, TMT-B and Corsi task; rotated sum of squared factor loadings explaining 17.56% variance), the second one consists mainly of language performance (eigenvalue = 1.32; highest rotated factor loadings on all subtests of the RWT and the MWTB; rotated sum of squared factor loadings explaining 10.82% variance). All rotated factor loadings on factor attention/working memory and factor language are shown in Table S4.

### Bivariate partial correlations gyrification and neuropsychological factors

We found several significant correlations between the extracted neuropsychological factors and mean gyrification cluster values from the conducted multiple regression (Table 3). In particular, the right superior frontal gyrus was related to language in the whole sample and the left supramarginal gyrus to both, working memory and language in the preterm subsample (28–38 weeks), among others. In this partial correlation analysis, gestational age in weeks was not associated with the neuropsychological factors (Table 3). Correlations between the neuropsychological factors gained from the factor analysis and gyrification clusters from *t*-tests showed similar results and are provided in the supplement (Table S5).

**Table 3:** Partial correlations between extracted mean gyrification clusters and neuropsychological factors, controlling for years of education

	Total		Gestational age 28–37 weeks (*n* = 51)		Gestational age ≥ 38 weeks (*n* = 491)	
	Working memory/attention	Language	Working memory/attention	Language	Working memory/attention	Language
Gestational age in weeks	− 0.021	− 0.059	− 0.076	− 0.17	-0.028	− 0.057
Left superior frontal	0.044	− 0.052	0.334*	− 0.014	0.01	− 0.054
Left lingual	− 0.025	− 0.082	− 0.007	− 0.089	− 0.027	− 0.078
Left supramarginal	− 0.032	0.043	0.321*	0.303*	− 0.071	0.017
Right lingual	0.014	− 0.05	0.154	− 0.149	0.001	− 0.039
Right superior frontal	0.062	–0.115**	0.218	− 0.104	0.046	− 0.114*

*p < 0.05, **p < 0.01

### Moderation analysis

**Fig. 2:**
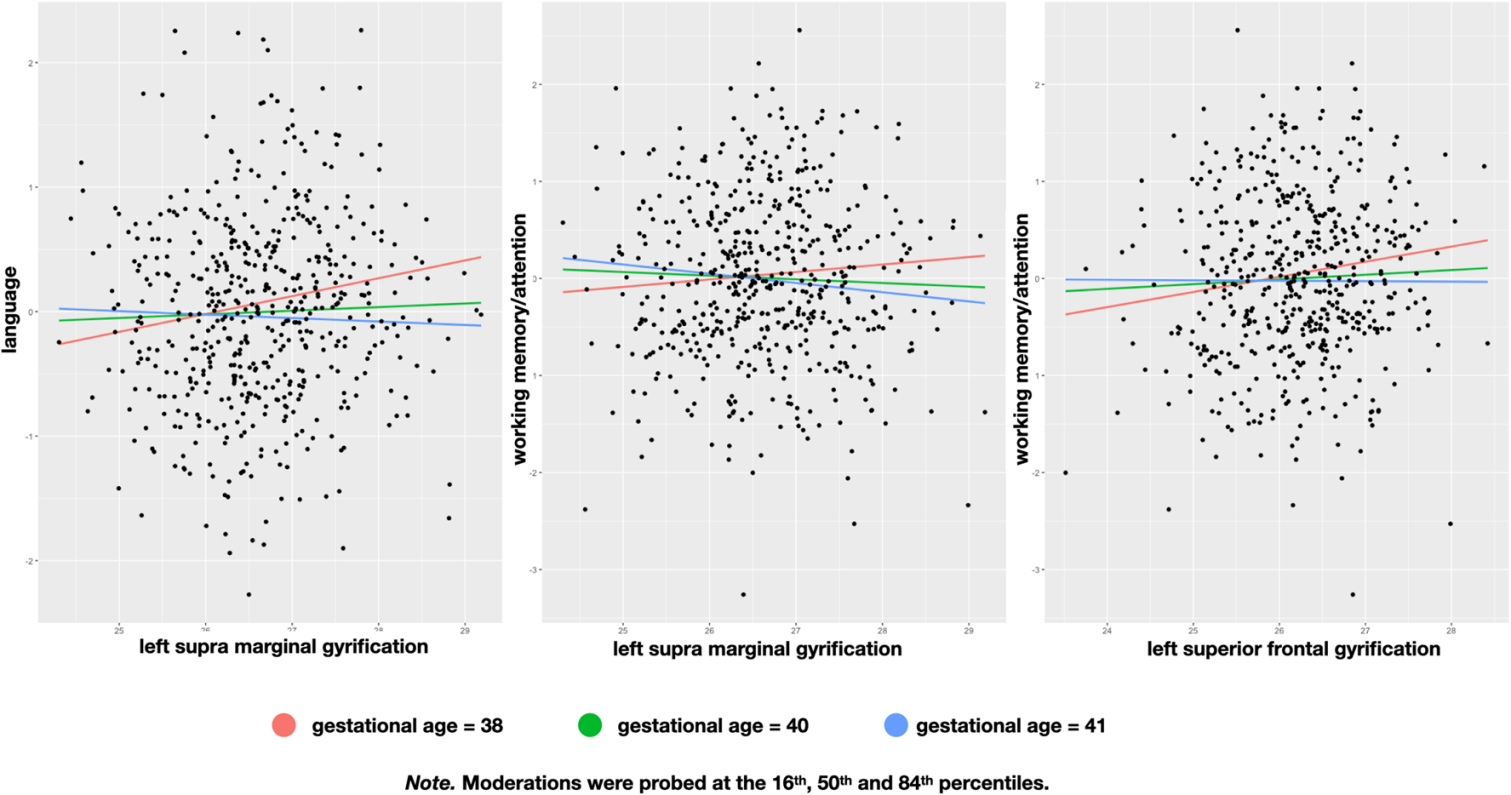
Linking gyrification, gestational age and neuropsychological factors language and working memory/attention. Effects from significant gyrification clusters on language and working memory/attention are significantly moderated by gestational age while controlling for years education

In three models, gestational age significantly moderated the association between mean gyrification cluster values extracted from significant clusters in the multiple regressions and neuropsychological factors while controlling for years of education (s. Fig. 2 and Table 4). Gestational age significantly moderated the association between the left supramarginal gyrification cluster and the factor working memory/attention (model: *F*(4/525) = 4.886, *p* = 0.001, *R*^*2*^ = 3.4%; moderation: *t* = − 2, *p* = 0.047, *R*^*2*^(change) = 0.8%) as well as the association between the same gyrification cluster and the factor language (model: *F*(4/525) = 4.015, *p* = 0.003, *R*^*2*^ = 3.1%; moderation: *t* = − 2.19*, p* = 0.029, *R*^*2*^(change) = 1%). Additionally, the association between the left superior frontal gyrification cluster and the factor working memory/attention was significantly moderated by gestational age (model: *F*(4/525) = 4.892, *p* = 0.001, *R*^*2*^ = 3.4%; moderation: *t* = − 2.04, *p* = 0.041, *R*^*2*^(change) = 0.7%). In summary, gestational age impacted on local gyrification which interacted again with gestational age on neuropsychological performance in adulthood in specific cognitive domains, which have previously been related to these cortical regions.

**Table 4:** Moderation analysis

	*F*(HC3)	*df*1*/df*2	*p*	*R*^*2*^	Coefficient	se(HC3)	*t*	*p*
Constant					− 0.743	0.208	− 3.57	< 0.001
Moderator: Gestational age					− 0.027	0.026	− 1.06	0.289
Dependent variable: Left superior frontal					0.054	0.051	1.06	0.29
Interaction					− 0.058	0.028	− 2.04	0.041
Covariate: Years of education					0.055	0.015	3.73	< 0.001
*Model summary* *Outcome: working memory/attention*	4.892	4/525	0.001	0.034				
Constant					− 0.56	0.198	− 2.83	0.005
Moderator: Gestational age					− 0.024	0.022	− 1.1	0.271
Dependent variable: Left superior frontal					− 0.043	0.047	− 0.92	0.356
Interaction					0.001	0.026	0.02	0.981
Covariate: Years of education					0.04	0.014	2.85	0.005
*Model summary* *Outcome: language*	2.608	4/525	0.035	0.02				
Constant					− 0.749	0.208	− 3.61	< 0.001
Moderator: Gestational age					− 0.018	0.025	− 0.71	0.476
Dependent variable: Left supramarginal					− 0.028	0.045	− 0.64	0.524
Interaction					− 0.057	0.029	− 2	0.047
Covariate: Years of education					0.055	0.015	3.78	< 0.001
*Model summary* *Outcome: working memory/attention*	4.886	4/525	0.001	0.034				
Constant					− 0.511	0.197	− 2.6	0.01
Moderator: Gestational age					− 0.04	0.02	− 2	0.046
Dependent variable: Left supramarginal					0.046	0.037	1.26	0.221
Interaction					− 0.056	0.026	− 2.19	0.029
Covariate: Years of education					0.038	0.014	2.69	0.007
*Model summary* *Outcome: language*	4.015	4/525	0.003	0.031				
Constant					− 0.75	0.206	− 3.64	< 0.001
Moderator: Gestational age					− 0.018	0.023	− 0.76	0.447
Dependent variable: Right superior frontal					0.061	0.043	1.41	0.158
Interaction					− 0.006	0.037	− 0.15	0.878
Covariate: Years of education					0.054	0.015	3.75	< 0.001
*Model summary* *Outcome: working memory/attention*	4.069	4/525	0.003	0.029				
Constant					− 0.557	0.196	− 2.84	0.005
Moderator: Gestational age					− 0.019	0.024	− 0.79	0.433
Dependent variable: Right superior frontal					− 0.088	0.037	− 2.37	0.018
Interaction					0.002	0.024	0.06	0.947
Covariate: Years of education					0.04	0.014	2.86	0.004
*Model summary* *Outcome: language*	3.54	4/525	0.007	0.029				
Constant					− 0.75	0.208	− 3.64	< 0.001
Moderator: Gestational age					− 0.007	0.029	− 0.23	0.817
Dependent variable: Left lingual					− 0.023	0.047	− 0.49	0.622
Interaction					0.005	0.026	0.19	0.848
Covariate: Years of education					0.055	0.015	3.73	< 0.001
*Model summary* *Outcome: working memory/attention*	3.67	4/525	0.006	0.025				
Constant					− 0.551	0.198	− 2.78	0.006
Moderator: Gestational age					− 0.017	0.023	− 0.73	0.466
Dependent variable: Left lingual					− 0.072	0.046	− 1.56	0.12
Interaction					0.01	0.028	0.36	0.718
Covariate: Years of education					0.03	0.014	2.81	0.005
*Model summary* *Outcome: language*	2.935	4/525	0.02	0.024				
Constant					− 0.741	0.207	− 3.57	< 0.001
Moderator: Gestational age					− 0.021	0.029	− 0.73	0.465
Dependent variable: Right lingual					0.019	0.041	0.04	0.64
Interaction					− 0.04	0.034	− 1.19	0.234
Covariate: Years of education					0.054	0.015	3.74	< 0.001
*Model summary* *Outcome: working memory/attention*	3.877	4/525	0.004	0.029				
Constant					− 0.535	0.198	− 2.70	0.007
Moderator: Gestational age					− 0.03	0.024	− 1.26	0.208
Dependent variable: Right lingual					− 0.031	0.035	− 0.87	0.383
Interaction					− 0.025	0.023	− 1.06	0.288
Covariate: Years of education					0.039	0.014	2.78	0.006
*Model summary* *Outcome: language*	2.96	4/525	0.019	0.022				

Heteroscedasticity consistent standard error and covariance matrix estimator was used (HC3) [85, 86]. Continuous variables that define products were mean centered prior analysis

## Discussion

We investigated associations between birth weight, gestational age and gyrification in a large sample of adults, excluding high risk pregnancies and births. We then explored the impact of these associations on neurocognition. We found a positive linear association between gestational age and gyrification bilaterally in the superior frontal cortex, the left supramarginal cortex and in the lingual cortex bilaterally. The association between gyrification clusters and the neuropsychological factors language and working memory/attention was moderated by gestational age. These findings provide an important basis for understanding prenatal influences on brain morphology and their relations to cognitive functions in healthy adults.

We demonstrate positive, linear associations between duration of pregnancy and gyrification in healthy adults. We extracted gyrification values using an absolute mean curvature approach which is positively correlated with increases in total surface area [64]. A methodological strength of this study is the exclusion of participants whose mothers had high-risk pregnancies (e.g., infections, malnutrition, drug abuse, etc.; see above) or pregnancies that are known to be associated with aberrant cortical folding in the fetuses (i.e., multiple pregnancy). Due to this approach, we can conclude that strong associations between rather subtle environmental influences (measured as gestational age) and cortical formation evidently persist in adults even when excluding more severe environmental effects during pregnancy that could have confounded these results. These findings emphasize the formative character from gestational age on brain anatomy which can already be observed in new-borns [87] and shed new light on the importance of preserving maternal health during pregnancy.

Our findings can be interpreted in several ways. First, prenatal cortical growth that is interrupted due to early birth might postnatally not be completed because cortical maturation could potentially only be completed in utero. Second, postnatal compensatory mechanisms of cortical maturation could fail because the harming cause (e.g., maternal stress [15, 88] or smoking [89, 90]) that lead to reduced gestational age still persists in postnatal environment. The third explanation comes from a core assumption of the concept of Developmental Origins of Health and Disease [7, 91, 92]. It posits prenatal mechanisms of permanent fetal programming that are triggered by in utero environmental effects that result in persistent modifications on the epigenome of the differentiating brain cells, which can lead to permanent physiological modifications in the offspring [15]. The consequences of this in utero adaption are twofold: On the one hand, these altered cortical formations are due to the plasticity of the fetal brain which prepares the unborn for its postnatal environment—assuming it is similar to its environment in utero [93]. Thereby, chances of immediate survival after birth and potential reproduction are maximized [94]. On the other hand, these effects can also result in non-adaptive physiology when there is a mismatch between prenatal adaptive brain development and the biological demands that the postnatal environment places on the new-born child. This maladjustment could then lead to vulnerability for later mental disorders.

Our second main result is that gestational age moderates the relationship between localized gyrification and the neuropsychological factors language and working memory/attention. The association between a cluster mostly comprising the left superior frontal cortex and the factor working memory/attention was moderated by gestational age. The involvement of the superior frontal gyrus in working memory has been demonstrated consistently [95]. Additionally, relationships between a gyrification cluster in the left supramarginal gyrus and the factor language as well as the factor working memory/attention were both again moderated by gestational duration. The supramarginal gyrus, as part of Wernicke’s area, has repeatedly been linked to phonological decisions [96, 97], syntax [98] and semantics [99, 100]. Another line of research showed that the supramarginal gyrus is involved in verbal/auditory working memory [101, 102]. Therefore, our results could demonstrate that variations in working memory and language performance are to a certain extent a product of the interactional effect of gestational age and gyrification alterations that are linked to prenatal cortical development. These findings are an important contribution to the identification of neurobiological pathways involved in the association between preterm birth and lower cognitive performance in adults that have been reported in epidemiological studies [4].

Since we found only one direct bivariate association between a gyrification cluster significantly associated with gestational age and one neuropsychological factor but moderated effects it should be pointed out that alterations in gyrification alone might not be sufficient to explain some aspects of poorer neuropsychological outcome. Rather, it is an interplay between variation in gyrification and other neurobiological factors that are influenced by gestational age. This highlights again the importance of determining relationships between prenatal and early-life factors influencing cognitive ability in adulthood.

We found no brain morphological associations with birth weight when we applied a strict statistic (FWE in SPM), although birth weight and gestational age were correlated (*r* = 0.378; *p* < 0.001). Gestational age might be a more valid proxy for brain maturation because birth weight is affected by many variables, such as maternal ethnicity, infant gender, maternal smoking and maternal diabetes [103].

Our findings also have implications for psychopathology. Cohort studies have shown that low gestational age is associated with a higher chance to develop a mental disorder in adulthood [104, 105]. Individuals who are at high risk of developing schizophrenia or who will later develop schizophrenia show general cognitive deficits in various domains before the onset of the disorder, including language, processing speed, working memory, executive functioning and intelligence [106, 107]. On a brain morphological level, there are gyrification differences between individuals with higher risk for schizophrenia and control subjects that partly overlap with cortical areas that were associated with gestational age in our study [108, 109]. We suggest that the interaction effect between gestational age and gyrification alterations associated with shortened gestational length may constitute a risk phenotype for mental disorders clinically characterized by reduced cognitive performance.

## Limitations and future studies

First, we asked our subjects to state their gestational age, birth weight and their mothers’ pregnancy courses using questionnaires and did not have their birth certificates available. Second, we used both birth weight and gestational age as a summary proxy for the success of prenatal neurodevelopment which is affected by a range of different materno-fetal factors. Future studies should also include patients in their samples to investigate potential interactive effects between gestational duration and pathogenic factors on gyrification as well as cognition.

## Conclusion

This study links aberrant local gyrification associated with gestational age to particular neuropsychological domains in healthy adults. We show positive associations between the duration of pregnancy and gyrification alterations in the superior frontal, lingual, and left supramarginal cortex in healthy adults. Additionally, relationships between aberrant gyrification clusters and the factors working memory/attention and language were moderated by gestational age. Our findings demonstrate that effects of prenatal development are permanently present in adult gyrification and have interactional effects on specific cognitive domains. Consequently, our study expands on the importance of maternal health during pregnancy and functions as an impetus to further improve maternal health during pregnancy to preserve cognitive abilities in offspring.

## Supplementary Information

Below is the link to the electronic supplementary material.

Supplementary file1 (DOCX 2354 KB)

## Data Availability

Research data are not shared.

## Declarations

### Data access and responsibility

All PIs take responsibility for the integrity of the respective study data and their components. All authors and coauthors had full access to all study data.
