# Supplementary material for: Associations of gestational age with gyrification and neurocognition in healthy adults
Source: Eur Arch Psychiatry Clin Neurosci. 2022 Jul 29;273(2):467–79. doi: 10.1007/s00406-022-01454-0 (PMC10070217; doi:10.1007/s00406-022-01454-0)
Supplement: Supplementary file 1 — Supplementary file1 (DOCX 2354 KB) [file 406_2022_1454_MOESM1_ESM.docx]

**Supplement: Associations of gestational age with gyrification and neurocognition in healthy adults**

Simon Schmitt^1,2^, Kai G. Ringwald^1,2^, Tina Meller^1,2^, Frederike Stein^1,2^, Katharina Brosch^1,2,3^, Julia-Katharina Pfarr^1,2^, Tim Hahn^4^, Hannah Lemke^4^, Susanne Meinert^4^, Jonathan Repple^4^, Katharina Thiel^4^, Lena Waltemate^4^, Alexandra Winter^4^, Dominik Grotegerd^4^, Astrid Dempfle^6^, Andreas Jansen^1,2,3,5^, Axel Krug^7^, Udo Dannlowski^4^, Igor Nenadić^1,2,3^, Tilo Kircher^1,2,3^

^1^ Department of Psychiatry and Psychotherapy, Philipps-Universität Marburg, Rudolf-Bultmann-Str. 8, 35039 Marburg, Germany

^2^ Center for Mind, Brain and Behavior (CMBB), University of Marburg and Justus Liebig University Giessen, Hans-Meerwein-Str. 6, 35032 Marburg, Germany

^3^ Marburg University Hospital – UKGM, Rudolf-Bultmann-Str. 8, 35039 Marburg, Germany

^4^ Institute for Translational Psychiatry, University of Münster, Münster, Germany

^5^ Core-Facility BrainImaging, Faculty of Medicine, Rudolf-Bultmann-Str. 8, 35039 Marburg, Germany

^6^ Institute of Medical Informatics and Statistics, University Hospital Schleswig-Holstein, Kiel, Germany

^7^ Department of Psychiatry and Psychotherapy, University of Bonn, Bonn, Germany

Corresponding author:

Simon Schmitt

Department of Psychiatry and Psychotherapy, Philipps-Universität Marburg, Rudolf-Bultmann-Str. 8, 35039 Marburg, Germany

Phone: +49 6421 58 64454

Email: s.schmitt@staff.uni-marburg.de

[Supplementary methods 4](#_Toc106226414)

[1. Description of the neurocognitive measures 4](#_Toc106226415)

[1.1. VLMT 4](#_Toc106226416)

[1.2. Trailmaking test (TMT-B) 4](#_Toc106226417)

[1.3. The Corsi task 4](#_Toc106226418)

[1.4. Letter-number sequencing subtest 5](#_Toc106226419)

[1.5. Digit symbol substitution test (DSST) 5](#_Toc106226420)

[1.6. d2 test of attention 5](#_Toc106226421)

[1.7. Regensburger Wortflüssigkeits-Test (RWT) 6](#_Toc106226422)

[1.8. Multiple choice vocabulary test 6](#_Toc106226423)

[Supplemental tables 7](#_Toc106226424)

[Table S1 Anti-Image correlation matrix of neuropsychological test scores 7](#_Toc106226425)

[Table S2 Intercorrelations of neuropsychological tests 8](#_Toc106226426)

[Table S3 Significant higher gyrification in participants born after full-term pregnancy relative to those born preterm. 9](#_Toc106226427)

[Table S4 Rotated factor matrix 10](#_Toc106226428)

[Table S5 Partial correlations between extracted mean gyrification clusters and neuropsychological factors, controlling for years of education 11](#_Toc106226429)

[Supplemental figures 12](#_Toc106226430)

[Figure S1 Scatter plots of the associations between gestational age and gyrification 12](#_Toc106226431)

[Figure S2 Gyrification differences between individuals born preterm versus individuals born after full-term pregnancy 15](#_Toc106226432)

[Figure S3 Scree plot displaying the eigenvalues of components extracted by using principal axis factoring 16](#_Toc106226433)

[References 17](#_Toc106226434)

# Supplementary methods

## 1. Description of the neurocognitive measures

Neuropsychological tests were applied in the order in which they are listed here.

### 1.1. VLMT

We assessed declarative episodic memory using the German adaption of the Rey Auditory Verbal Learning Test (RAVLT; Bean, 2011), the verbal learning and memory test (VLMT; Helmstaedter & Durwen, 1990). In this test, five consecutive times, the same list consisting of 15 words, is read out by the instructor and the participant is asked to recall them directly afterwards. The sum of all items recalled correctly is a measurement of declarative short-term memory (VLMT-A). After a delay of 30 minutes and without representing the word list again, the participant is asked to recall it again (VLMT-B).

### 1.2. Trailmaking test (TMT-B)

Executive functioning and speed of cognitive processing was assessed with the Trail Making Test (TMT; Bowie & Harvey, 2006; Sánchez-Cubillo et al., 2009). In this paper-pencil-test, participants have to connect numbers ascendingly and letter alphabetically in alternating fashion. The time to complete the task is measured in seconds.

### 1.3. The Corsi task

This test (Corsi, 1972) is included in the Wechsler Memory scale (Wechsler, 1997) and assesses the visuospatial short-term and working memory by using includes a small board to which eight small blocks are attached. During the first part of the test (Corsi A) participants are instructed to tap on the blocks in an order which was shown to them by the experimenter directly before. In the second part of the test (Corsi B) blocks are again tapped by the experimenter but subjects are asked to tap them in reverse order. The Corsi A consists of 14 trials, the Corsi B of 12, respectively. The sum of correct responses in both parts of the test is reported.

### 1.4. Letter-number sequencing subtest

The letter-number sequencing subtest (LNST) was taken from the Wechsler Adult Intelligence Scale (WAIS; Wechsler, 1997). It assesses the verbal capacity of the working memory. This test comprises 24 items consisting of different amounts of letters and numbers that are read out by the instructor. The participant is asked to rearrange the letters and numbers starting with numbers in ascending order and continuing with the letters in alphabetical order. The first item includes three letters and numbers, the later items are more difficult so that the maximum number of digits that have to be remembered, rearranged and reported is eight. The number of correctly answered items is recorded.

### 1.5. Digit symbol substitution test (DSST)

This German equivalent of the digit symbol substitution test (DSST) of the WAIS (Wechsler, 1997) is another paper-pencil-test that is used as an indicator of attention as well as cognitive and motor processing speed. At the beginning of the test nine different digit-symbol pairs are introduced. Then, a list with digits is presented and the participant is asked to add as many correct symbols to each digit as possible within 90 seconds.

### 1.6. d2 test of attention

Sustained and selective attention was measured with the d2 test of attention (Brickenkamp, Schmidt-Atzert, & Liepmann, 2010; Steinborn, Langner, Flehmig, & Huestegge, 2018). Participants have to discriminate between stimuli relevant for a task and disturbing ones. We used the concentration performance score (d2-KL) of the d2 test. This score is computed by summing up all items that were marked correctly by the participant and then subtracting the number of items marked incorrectly (F2) and the erroneously omitted symbols (F1).

### 1.7. Regensburger Wortflüssigkeits-Test (RWT)

In order to measure verbal fluency we used the Regensburger Wortflüssigkeits-Test (Aschenbrenner, Tucha, & Lange, 2000). Participants are asked to produce as many words of three different categories within the time of each 60 seconds. The first category is “animals” (RWT-A), the second one “words, that start with the letter ‘p’” (RWT-P) and the last category is alternating between fruits and sports (RWT-alt). All correct words are recorded (after subtraction of errors and repetitions).

### 1.8. Multiple choice vocabulary test

This test was applied to verbal IQ (Lehrl, 1995). It is the German equivalent of the National Adult Reading Test (NART; Nelson & Willison, 1991). The MWT-B (Mehrfachwahl-Wortschatztest, Version B) consists of 37 rows with each five words. Only one word in each row is a correct German word. Subjects are asked to mark this one. The test score consists of all correctly marked words.

# Supplemental tables

## Table S1 Anti-Image correlation matrix of neuropsychological test scores

|  | VLMT-A | VLMT-B | TMT-B | Corsi | LNST | DSST | d2-KL | RWT-A | RWT-P | RWT-alt | MWT-B |
| --- | --- | --- | --- | --- | --- | --- | --- | --- | --- | --- | --- |
| VLMT-A | 0.852 | 0.265 | 0.062 | -0.113 | -0.098 | -0.08 | -0.092 | -0.035 | -0.11 | -0.035 | 0.004 |
| VLMT-B | 0.265 | 0.71 | 0.047 | 0.104 | -0.021 | -0.027 | 0.044 | -0.008 | 0.014 | 0.035 | -0.032 |
| TMT-B | 0.062 | 0.047 | 0.883 | 0.119 | 0.101 | 0.237 | 0.134 | 0.049 | 0.01 | 0.033 | 0.024 |
| Corsi | -0.113 | 0.104 | 0.119 | 0.863 | -0.239 | -0.133 | -0.099 | -0.04 | -0.036 | 0.05 | 0.014 |
| LNST | -0.098 | -0.021 | 0.101 | -0.239 | 0.852 | 0.024 | -0.122 | -0.018 | -0.069 | -0.065 | -0.123 |
| DSST | -0.08 | -0.027 | 0.237 | -0.133 | 0.024 | 0.796 | -0.404 | -0.079 | -0.036 | -0.126 | 0.119 |
| d2-KL | -0.092 | 0.044 | 0.134 | -0.099 | -0.122 | -0.404 | 0.826 | -0.046 | 0.046 | -0.047 | -0.026 |
| RWT-A | -0.035 | -0.008 | 0.049 | -0.04 | -0.018 | -0.079 | -0.046 | 0.822 | -0.237 | -0.262 | -0.112 |
| RWT-P | -0.11 | 0.014 | 0.01 | -0.036 | -0.069 | -0.036 | 0.046 | -0.237 | 0.822 | -0.107 | -0.125 |
| RWT-alt | -0.035 | 0.035 | 0.033 | 0.05 | -0.065 | -0.126 | -0.047 | -0.262 | -0.107 | 0.842 | -0.072 |
| MWT-B | 0.004 | -0.032 | 0.024 | 0.014 | -0.123 | 0.119 | -0.026 | -0.112 | -0.125 | -0.072 | 0.7 |

## Table S2 Intercorrelations of neuropsychological tests

|  | d2-KL | TMT-B | Corsi | DSST | RWT-A | RWT-P | RWT-Alt | LNST | VLMT-A | VLMT-B | MWT-B |
| --- | --- | --- | --- | --- | --- | --- | --- | --- | --- | --- | --- |
| d2-KL | — |  |  |  |  |  |  |  |  |  |  |
| TMT-B | -0.416** | — |  |  |  |  |  |  |  |  |  |
| Corsi | 0.373** | -0.346** | — |  |  |  |  |  |  |  |  |
| DSST | 0.569** | -0.467** | 0.387** | — |  |  |  |  |  |  |  |
| RWT-A | 0.256** | -0.242** | 0.206** | 0.288** | — |  |  |  |  |  |  |
| RWT-P | 0.145** | -0.158** | 0.174** | 0.188** | 0.359** | — |  |  |  |  |  |
| RWT-Alt | 0.236** | -0.214** | 0.137** | 0.289** | 0.381** | 0.287** | — |  |  |  |  |
| LNST | 0.329** | -0.298** | 0.382** | 0.265** | 0.216** | 0.205** | 0.199** | — |  |  |  |
| VLMT-A | 0.33** | -0.269** | 0.33** | 0.318** | 0.22** | 0.222** | 0.193** | 0.278** | — |  |  |
| VLMT-B | -0.122** | 0.063 | -0.178** | -0.087* | -0.049 | -0.085* | -0.067 | -0.081 | -0.25** | — |  |
| MWT-B | 0.064 | -0.072 | 0.059 | -0.006 | 0.2** | 0.204** | 0.15** | 0.177** | 0.064 | 0.01 | — |

* = significant at ⍺ = 0.05 (two-tailed), ** = significant at ⍺ = 0.01 (two-tailed).

## Table S3 Significant higher gyrification in participants born after full-term pregnancy relative to those born preterm.

| *k* | *TFCE* | *p_FWE_* | Coordinates |  | Anatomical region |
| --- | --- | --- | --- | --- | --- |
| 1057 | 15713.68 | 0.0141 | 7/3/61 | 59% | r. superior frontal |
|  |  |  |  | 19% | r. posterior cingulate |
|  |  |  |  | 16% | r. paracentral |
|  |  |  |  | 5% | r. caudal anterior cingulate |
|  |  |  |  |  |  |
| 7591 | 15199.91 | 0.016 | -21/19/43 | 24% | l. superior frontal |
|  | 15161.68 | 0.0162 | -44/-29/50 | 16% | l. supramarginal |
|  | 15091.7 | 0.0165 | -36/-33/48 | 12% | l. superior parietal |
|  |  |  |  | 11% | l. postcentral |
|  |  |  |  | 9% | l. precentral |
|  |  |  |  | 5% | l. caudal middle frontal |
|  |  |  |  | 5% | l. bank of the superior temporal sulcus |
|  |  |  |  | 5% | l. posterior cingulate |
|  |  |  |  | 4% | l. rostral middle frontal |
|  |  |  |  | 3% | l. inferior parietal |
|  |  |  |  | 2% | l. caudal anterior cingulate |
|  |  |  |  | 2% | l. middle temporal |

*Note*. Correction for multiple testing was carried out with the family wise error rate (FWE) at α=0.05. Cluster labelling was conducted with the Desikan-Killiany atlas (Desikan et al., 2006).

## Table S4 Rotated factor matrix

| test | Factor-loadings factor attention/working memory | Factor-loadings factor language |
| --- | --- | --- |
| VLMT-A | 0.283 | 0.182 |
| VLMT-B | <0.1 | <0.1 |
| TMT-B | -0.562 | -0.163 |
| Corsi | 0.443 | 0.129 |
| LNST | 0.337 | 0.258 |
| DSST | 0.77 | 0.128 |
| d2-KL | 0.688 | 0.122 |
| RWT-A | 0.277 | 0.616 |
| RWT-P | 0.113 | 0.525 |
| RWT-Alt | 0.277 | 0.478 |
| MWT-B | <0.1 | 0.363 |

*Note.* Extraction method: principal axis factoring. Rotation method: Varimax with Kaiser normalization (rotation converged in 17 iterations).

## Table S5 Partial correlations between extracted mean gyrification clusters and neuropsychological factors, controlling for years of education

|  | total | |  | gestational age 28 - 37 weeks  (*n* = 51) | |  | gestational age ≥ 38 weeks  (*n* = 491) | |
| --- | --- | --- | --- | --- | --- | --- | --- | --- |
|  | *Working memory/ attention* | *language* |  | *Working memory/attention* | *language* |  | *Working memory/attention* | *language* |
| gestational age in weeks | -0.021 | -0.059 |  | -0.076 | -0.17 |  | -0.028 | -0.057 |
| Cluster l. hemisphere | 0.022 | 0.023 |  | 0.312* (*p* = 0.31) | 0.244 |  | -0.013 | 0.001 |
| Cluster r. hemisphere | 0.021 | -0.119** (*p* = 0.006) |  | 0.086 | -0.129 |  | 0.012 | -0.12** (*p* = 0.009) |

*Note*. * *p* <0.05, ** *p* <0.01

# Supplemental figures

## Figure S1 Scatter plots of the associations between gestational age and gyrification


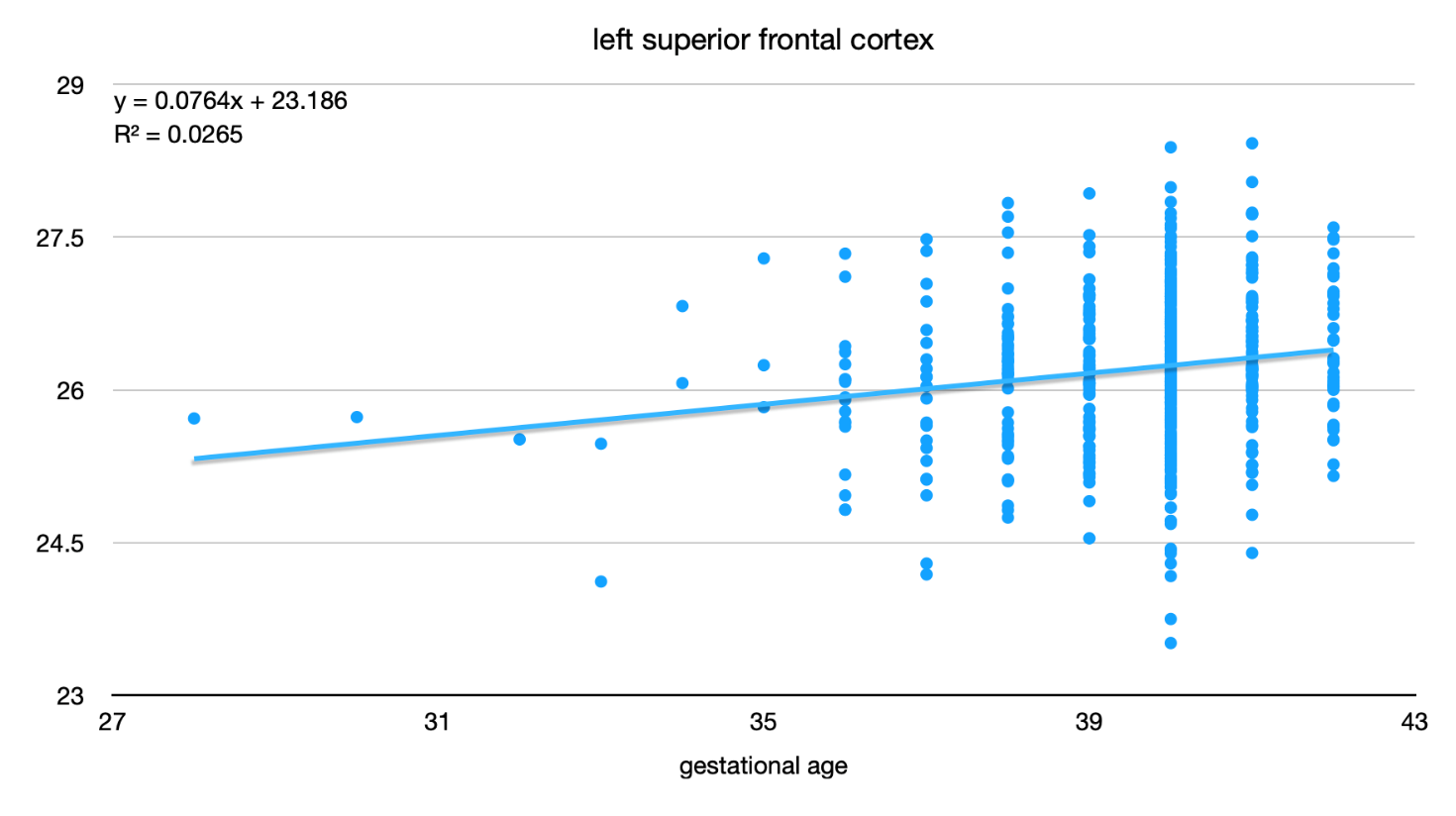

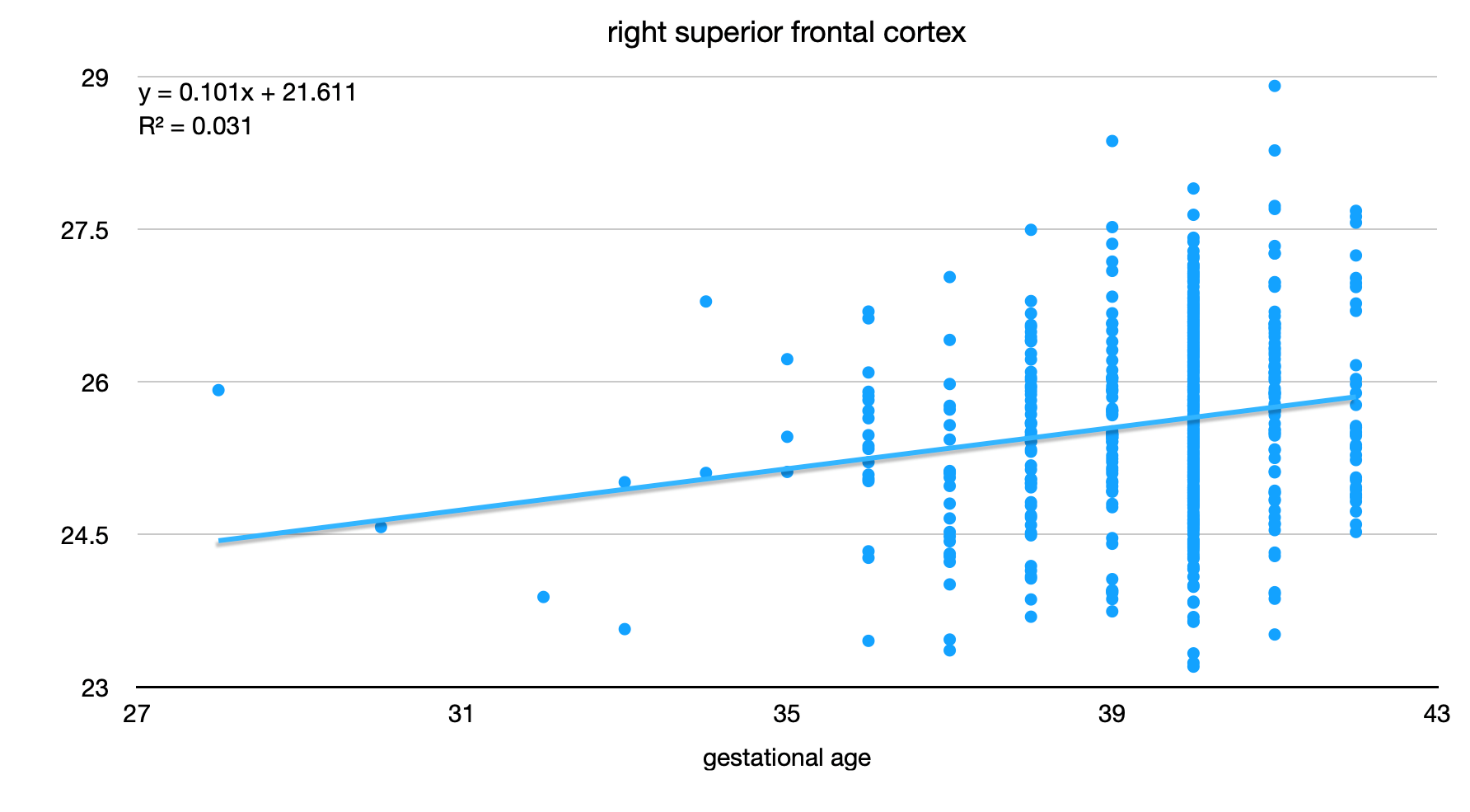

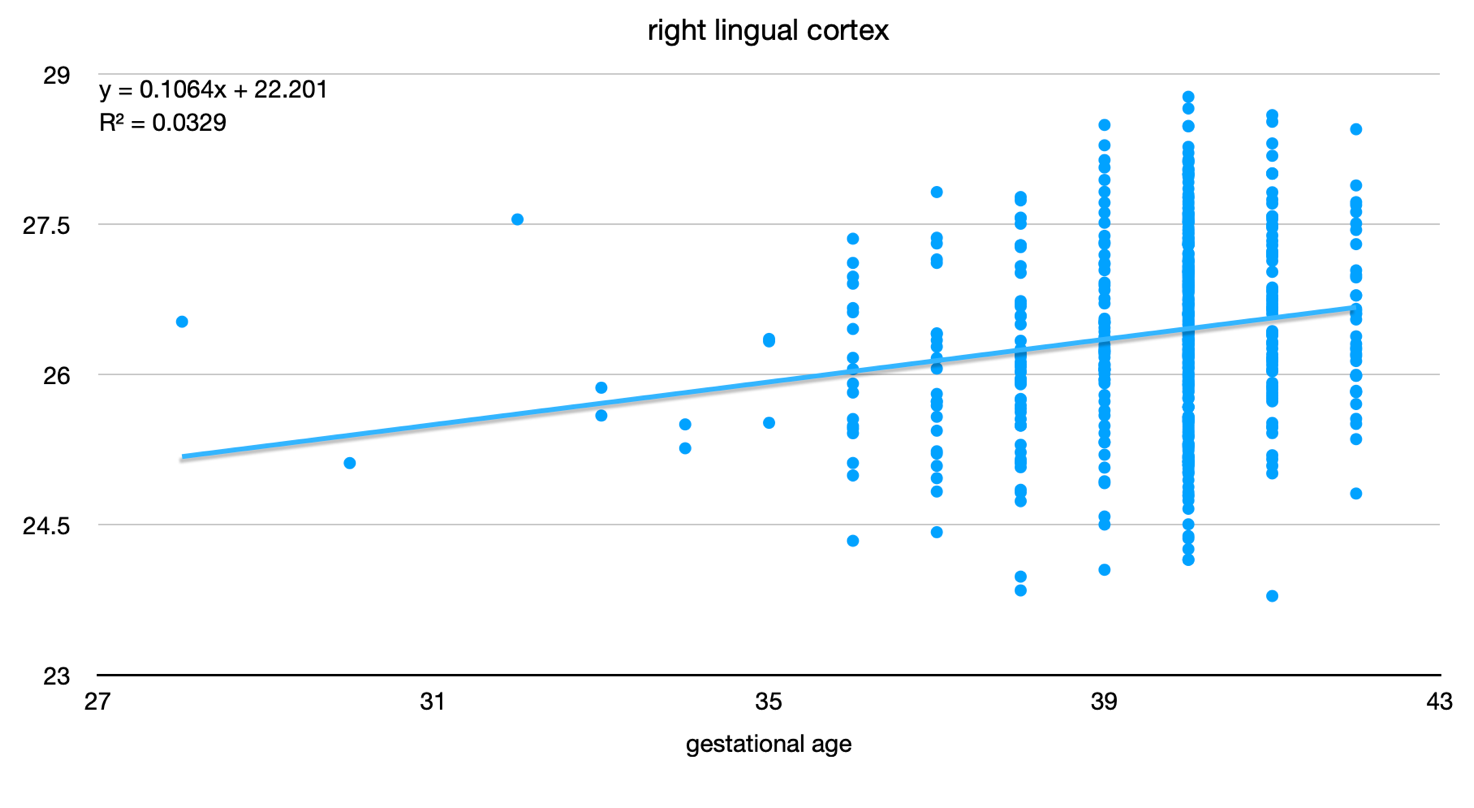

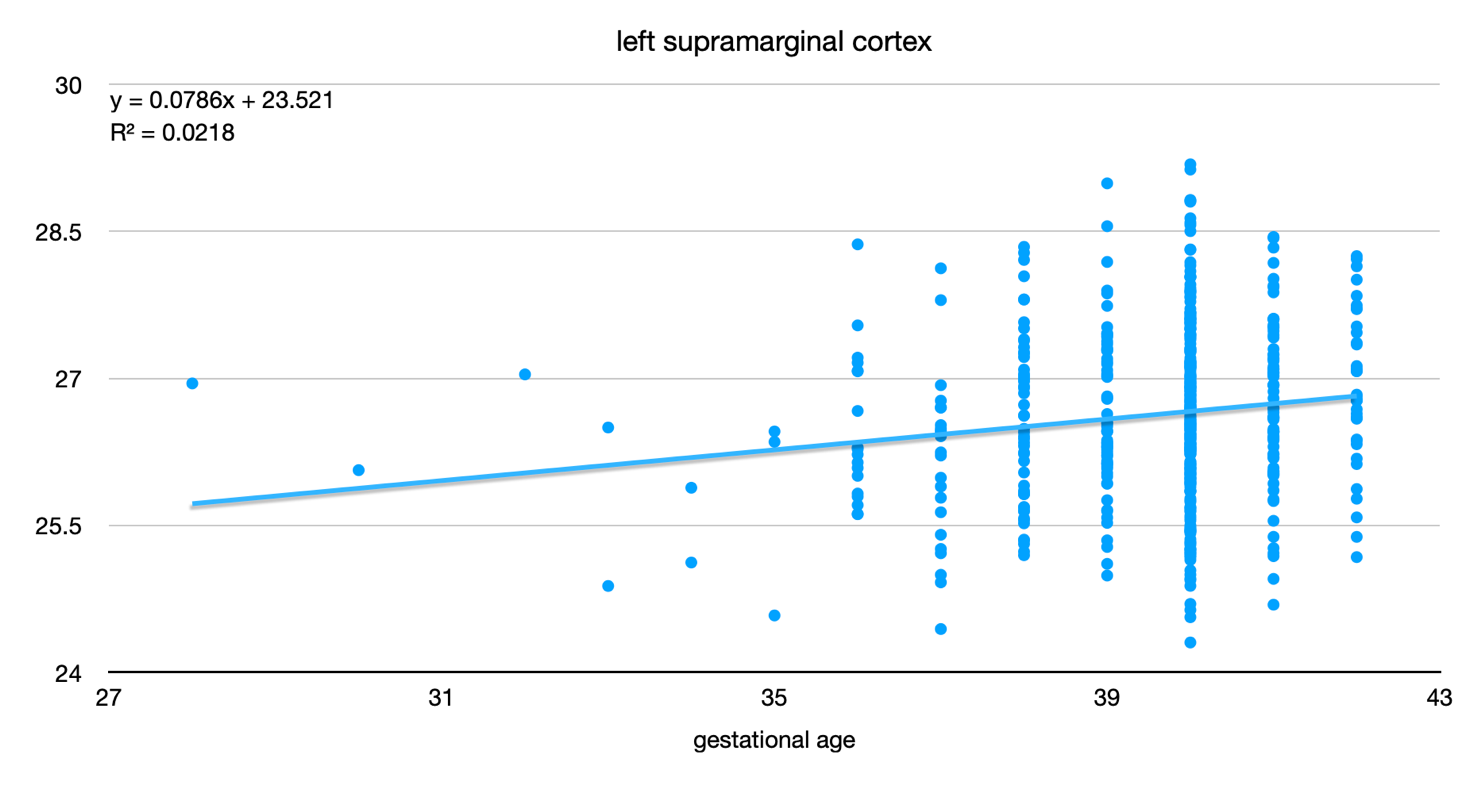

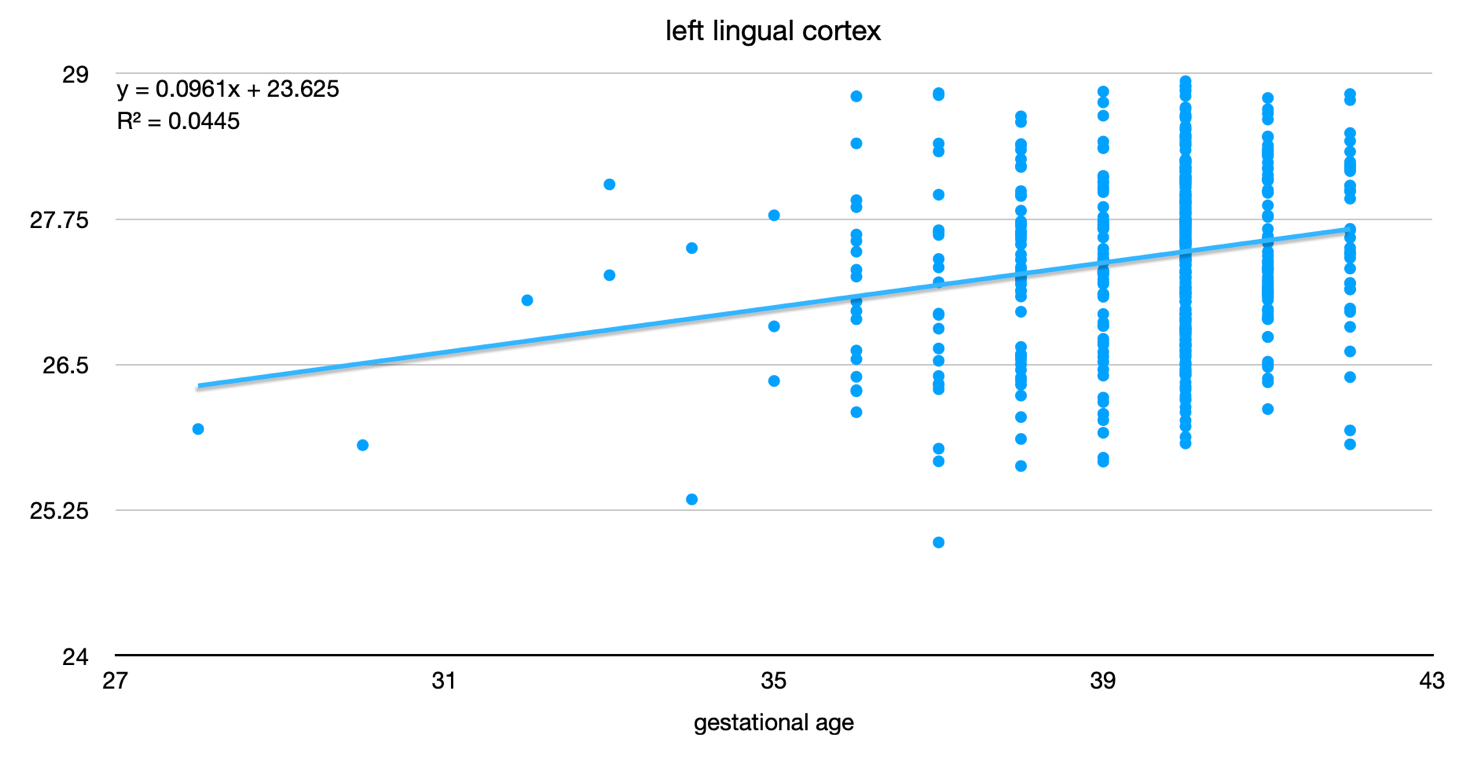


*Note.* When excluding outliers (defined as three interquartile ranges below the first quartile or above the third quartile), all gyrification clusters remained highly significantly associated with gestational age (in weeks): *r*(_left superior frontral.gestational age_)=0.15, *p*<0.001; *r*(_right superior frontal.gestational age_)=0.17, *p*<0.001; *r*(_right lingual.gestational age_)=0.19, *p*<0.001; *r*(_left supramarginal.gestational age_)=0.127, *p*=0.003; *r*(_left lingual.gestational age_)=0.17, *p*<0.001.

Figure S2 Gyrification differences between individuals born preterm versus individuals born after full-term pregnancy
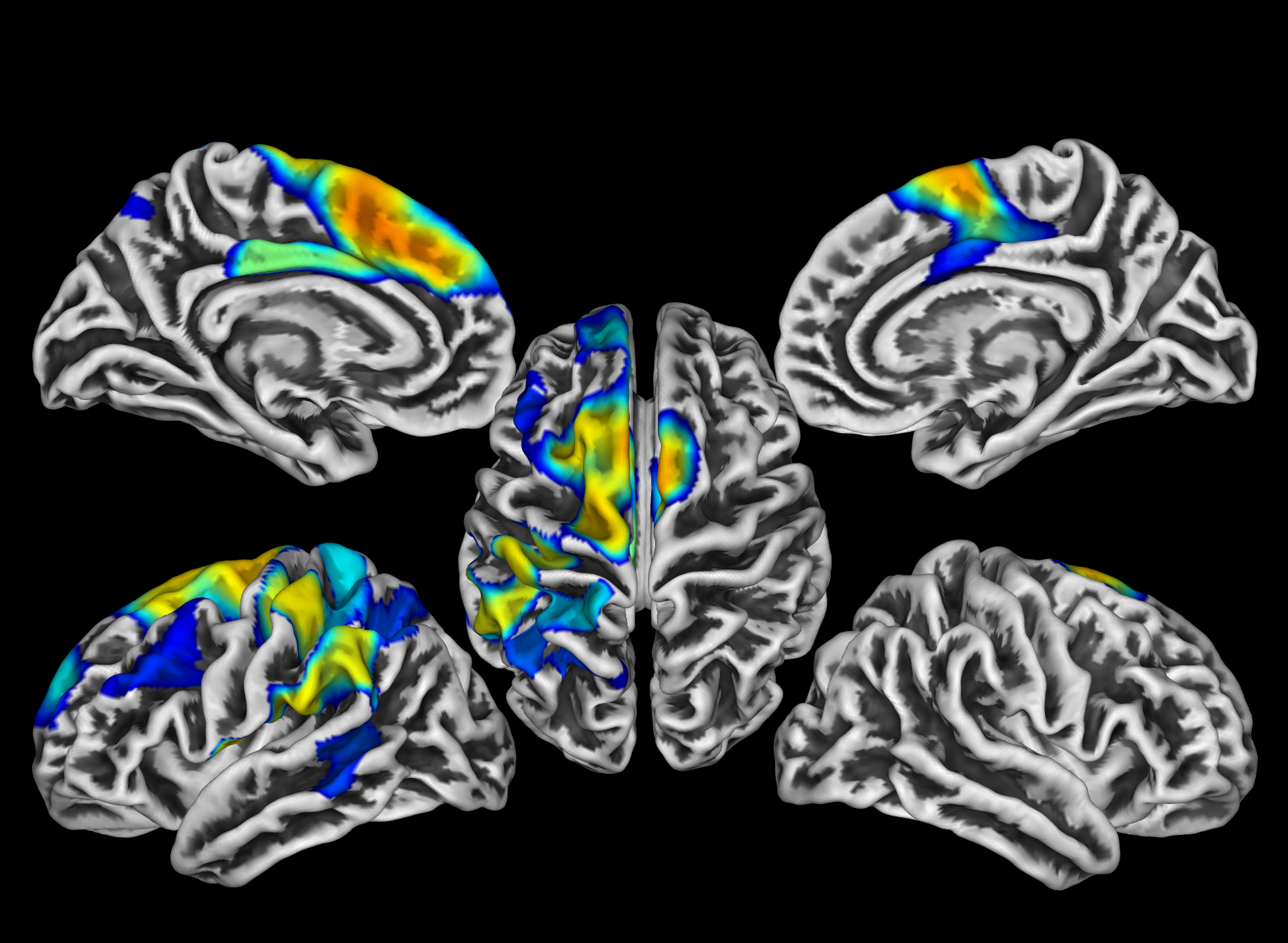


*Note.* Statistical parametric map of p-values derived from a t-test investigating relatively higher gyrification in individuals born preterm compared to individuals born after full-term pregnancy. Threshold-free cluster enhancement was used at a threshold of *p*<0.05 (FWE-corrected). Warmer colours represent lower p-values.

## Figure S3 Scree plot displaying the eigenvalues of components extracted by using principal axis factoring


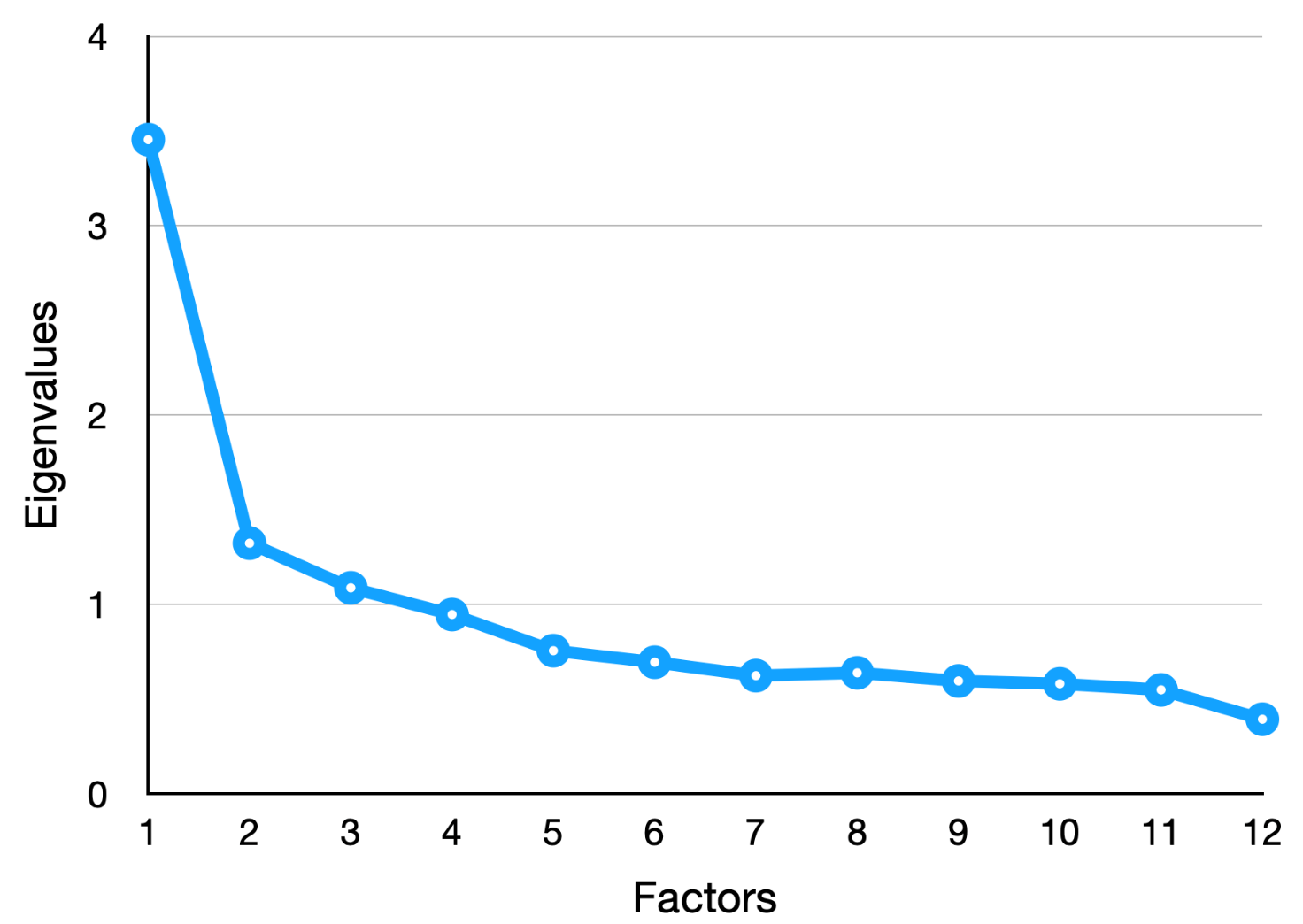


# References

Aschenbrenner, S., Tucha, O., & Lange, K. W. (2000). *Regensburger Wortflüssigkeits-Test: RWT*: Hogrefe, Verlag für Psychologie.

Bean, J. (2011). Rey Auditory Verbal Learning Test, Rey AVLT. In J. S. Kreutzer, J. DeLuca, & B. Caplan (Eds.), *Encyclopedia of Clinical Neuropsychology* (pp. 2174-2175). New York, NY: Springer New York.

Bowie, C. R., & Harvey, P. D. (2006). Administration and interpretation of the Trail Making Test. *Nature protocols, 1*(5), 2277.

Brickenkamp, R., Schmidt-Atzert, L., & Liepmann, D. (2010). *Test d2 - Revision Aufmerksamkeits und Konzentrationstest (Manual)*. Göttingen: Hogrefe.

Corsi, P. (1972). Memory and the medial temporal region of the brain. *Unpublished doctoral dissertation), McGill University, Montreal, QB*.

Desikan, R. S., Ségonne, F., Fischl, B., Quinn, B. T., Dickerson, B. C., Blacker, D., . . . Hyman, B. T. J. N. (2006). An automated labeling system for subdividing the human cerebral cortex on MRI scans into gyral based regions of interest. *NeuroImage, 31*(3), 968-980.

Helmstaedter, C., & Durwen, H. (1990). VLMT: Verbaler Lern-und Merkfähigkeitstest: Ein praktikables und differenziertes Instrumentarium zur Prüfung der verbalen Gedächtnisleistungen. *Schweizer Archiv für Neurologie, Neurochirurgie und Psychiatrie*.

Lehrl, S. (1995). *Mehrfachwahl-Wortschatz-Intelligenztest: MWT-B*. Göttingen: Hogrefe.

Nelson, H. E., & Willison, J. (1991). *National adult reading test (NART)*: Nfer-Nelson Windsor.

Sánchez-Cubillo, I., Periáñez, J., Adrover-Roig, D., Rodríguez-Sánchez, J., Rios-Lago, M., Tirapu, J., & Barcelo, F. (2009). Construct validity of the Trail Making Test: role of task-switching, working memory, inhibition/interference control, and visuomotor abilities. *Journal of the International Neuropsychological Society: JINS, 15*(3), 438.

Steinborn, M. B., Langner, R., Flehmig, H. C., & Huestegge, L. (2018). Methodology of performance scoring in the d2 sustained-attention test: Cumulative-reliability functions and practical guidelines. *Psychological Assessment, 30*(3), 339. doi:<https://doi.org/10.1037/pas0000482>

Wechsler, D. (1997). *WAiS-iii*: Psychological Corporation San Antonio, TX.
